# BRIDGING THE PRACTICE-RESEARCH DIVIDE: BUILDING SUCCESSFUL PARTNERSHIPS WITH STATE GOVERNMENT

**DOI:** 10.1093/geroni/igad104.1901

**Published:** 2023-12-21

**Authors:** Joan Davitt, Sol Baik, Joan Davitt

## Abstract

State governments collect data on LTSS but frequently lack resources to analyze it. Simultaneously, researchers often struggle to translate research to policy and practice realms. This symposium will elucidate strategies and challenges in building successful relationships between researchers and state government to enhance LTSS. Paper 1 explores the growing trend of states developing Multisector Plans for Aging (MPA) and opportunities for researchers to ensure that these state-led planning resources utilize evidence to transform and coordinate aging services across the state. This paper will discuss how states use data to promote accountability and monitor implementation of MPA initiatives. Paper 2 describes the development of a statewide data dashboard for Virginia that can be used by state and local agencies to leverage funding and improve programming. This paper will describe the process of developing and eliciting input from a stakeholder task force and the dashboard design. Paper 3 explores efforts in Minnesota to capitalize on existing data to inform policy advocacy and regulatory reform in assisted living. Data from annual survey inspections is utilized to explore trends, target education, and recommend policy changes. Paper 4 focuses on building relationships with Maryland’s Adult Protective Service division to engage collaboratively in logic modelling and continuous quality improvement. Strategies for translating existing administrative data into research ready formats and primary data collection methods will be discussed. Symposium participants will have an opportunity to discuss effective strategies for engaging state government in research.

